# Strategies to Improve Emergency Transitions From Long-Term Care Facilities: A Scoping Review

**DOI:** 10.1093/geront/gnae036

**Published:** 2024-04-25

**Authors:** Kaitlyn Tate, Greta Cummings, Frode Jacobsen, Gayle Halas, Graziella Van den Bergh, Rashmi Devkota, Shovana Shrestha, Malcolm Doupe

**Affiliations:** Faculty of Nursing, University of Alberta, Edmonton, Alberta, Canada; Faculty of Nursing, University of Alberta, Edmonton, Alberta, Canada; Centre for Care Research, Western Norway University of Applied Sciences, Bergen, Vestland, Norway; School of Dental Hygiene, University of Manitoba, Winnipeg, Manitoba, Canada; Department of Health and Functioning, Western Norway University of Applied Sciences, Bergen, Vestland, Norway; Faculty of Nursing, University of Alberta, Edmonton, Alberta, Canada; Faculty of Nursing, University of Alberta, Edmonton, Alberta, Canada; Rady Faculty Medicine, University of Manitoba, Winnipeg, Manitoba, Canada

**Keywords:** Emergency department, Long-term care, Nursing homes, Older adults, Transitions

## Abstract

**Background and Objectives:**

Older adults residing in residential aged care facilities (RACFs) often experience substandard transitions to emergency departments (EDs) through rationed and delayed ED care. We aimed to identify research describing interventions to improve transitions from RACFs to EDs.

**Research Design and Methods:**

In our scoping review, we included English language articles that (a) examined an intervention to improve transitions from RACF to EDs; and (b) focused on older adults (≥65 years). We employed content analysis. Dy et al.’s Care Transitions Framework was used to assess the contextualization of interventions and measurement of implementation success.

**Results:**

Interventions in 28 studies included geriatric assessment or outreach services (*n* = 7), standardized documentation forms (*n* = 6), models of care to improve transitions from RACFs to EDs (*n* = 6), telehealth services (*n* = 3), nurse-led care coordination programs (*n* = 2), acute-care geriatric departments (*n* = 2), an extended paramedicine program (*n* = 1), and a web-based referral system (*n* = 1). Many studies (*n* = 17) did not define what “improvement” entailed and instead assessed documentation strategies and distal outcomes (e.g., hospital admission rates, length of stay). Few authors reported how they contextualized interventions to align with care environments and/or evaluated implementation success. Few studies included clinician perspectives and no study examined resident- or family/friend caregiver-reported outcomes.

**Discussion and Implications:**

Mixed or nonsignificant results prevent us from recommending (or discouraging) any interventions. Given the complexity of these transitions and the need to create sustainable improvement strategies, future research should describe strategies used to embed innovations in care contexts and to measure both implementation and intervention success.

## Background and Objectives

As life expectancy increases, older persons (≥65 years) are more likely to live with multiple comorbid conditions often combined with functional and/or cognitive impairments (Sternberg et al., 2011). Older Canadians comprise about 20 to 40% of emergency department (ED) visits and their ED use rates have more than doubled in the last decade (Canadian Institute for Health Information, 2023). Transitions for older persons from residential aged care facilities (RACFs) to the ED are challenging as many of these individuals have complex needs are grappling with personal end-of-life decisions, and often lack the capacity to self-advocate their wishes (Aaltonen et al., 2014; Adamczyk et al., 2018; Cummings et al., 2012, 2020; Orth et al., 2021; Page et al., 2010). Older adults’ transitions from RACFs to the ED can result in poor outcomes related to functional decline, dehydration, infections, confusion, and loss of personal assistive devices (Cline, 2016; Cummings et al., 2020; Rantz et al., 2015; Trahan et al., 2016). For decades researchers have identified various substandard RACF to ED transitions for the frailest of our populations and have expressed an urgent need to improve these transitions (Adamczyk et al., 2018; Cummings et al., 2020; Tate et al., 2021, 2022, 2023). Challenges during transitions from RACFs to the ED include but are not limited to (a) difficulty coordinating care across various healthcare professionals from multiple care settings/cultures, (b) nonstandardized and insufficient documentation practices (Bruce & Suserud, 2005; Coleman et al., 2003; Morphet et al., 2014; Tate et al., 2023), (c) communication challenges that are exacerbated when older persons cannot clearly express their care needs (Page et al., 2010; Zimmerman et al., 2007), (d) atypical presentations when older people experience serious changes in health conditions (Armstrong, 2015; Chandra et al., 2015), and (e) ageism through rationing and delayed care (Adamczyk et al., 2018; Grief et al., 2013; Saif-Ur-Rahman et al., 2021; Van Wicklin. 2020).

Existing research on RACF to ED transitions focuses on knowledge creation (e.g., quantifies and presents factors associated with care transitions; Trahan et al., 2016), investigates strategies to *prevent* transitions from occurring (Cetin-Sahin et al., 2023; Grant et al., 2020), seeks to improve limited components of care (e.g., medication reconciliation; Chhabra et al., 2012; Grant et al., 2020), or describes staff perspectives and experiences of transitions (Chhabra et al., 2012; Gettel et al., 2020; LaMantia et al., 2016). Although most reviews examine traditional measures of intervention effectiveness (e.g., reduction in transfer rates), few authors have examined implementation processes simultaneously with the aforementioned measures (Proctor et al., 2011).

To the best of our knowledge, no authors have summarized the interventions tested to *improve* the RACF to ED care transition regardless of study design. The purpose of this review is to identify and describe the research examining interventions to improve transitions during RACF–ED transitions. We accepted study authors’ definitions or descriptions of improvement, or included studies in which no explicit definition was provided but where authors articulated an issue that occurred during these transitions that they aimed to address. We maintained this broad description of improvement to identify the various ways in which it is described or applied in the literature. Our objectives were to:

1. identify and describe interventions/strategies to improve transitions from RACF to the ED;
2. describe implementation strategies employed on interventions to improve transitions from RACF to the ED, such as how researchers embedded study interventions into local care contexts; and
3. identify approaches used to assess intervention and implementation effectiveness related to transition from RACF to the ED.

Our study objectives and analyses were guided by the Care Transitions Framework of Dy et al. (2015), which recognizes the complexity of healthcare environments and the need to ensure that interventions are properly embedded into these environments. This framework aligns with arguments put forth by Proctor et al. (2011) and Bowen et al. (2009) that fulsome evaluation of interventions in complex health systems requires diverse strategies that assess both traditional measures of intervention effectiveness (e.g., reduced transfer rates) and implementation success (e.g., stakeholder acceptability, feasibility, fidelity, sustainability of an intervention). In a collective effort to sustain intervention effectiveness in “real-life” settings, common themes across these and related frameworks emphasize the need to ensure that planned interventions are properly embedded into care environments, and that evaluation techniques assess the success of this contextualization along with intervention success. This approach also has value for differentiating poor intervention ideas from promising practices that require better implementation processes (Bauer & Kirchner, 2020).

## Research Design and Methods

We employed Levac et al.’s (2010) approach to conducting a scoping review. We used the PRISMA-ScR (Preferred Reporting Items for Systematic reviews and Meta-Analyses: extension for Scoping Reviews; Supplementary File 1) reporting guidelines (Moher et al., 2009).

### Inclusion and Exclusion Criteria

We included literature examining strategies to improve RACF–ED transitions. Detailed inclusion and exclusion criteria are available in Table 1.

**Table 1.** Inclusion and Exclusion Criteria for Literature on Improving Transitions From RACF to EDs

Criteria	Include	Exclude
Topic	Literature examining an intervention or strategy intended to improve transitions from RACFs (nursing homes/long-term care or supportive living/assisted living facilities) to the ED. We accepted author definitions of improved RACF to ED transitions and the measures they posed as operationalizing these definitions.	Literature examining interventions aimed at *only* preventing/reducing RACF to ED transitions. Literature on transitions from ED to RACF. Literature on RACF to ED transitions for planned procedures or elective treatment.
Population	Older adults (persons ≥65 years of age).	Individuals younger than 65 years.
Design or publication type	Primary research studies, secondary analyses, and gray literature.	Excluded published conference abstracts, editorials and systematic reviews.

*Notes:* ED = emergency department; RACF = residential aged care facility. We included only articles written in English and we did not place restrictions on year of publication.

### Search Strategy

We developed a search strategy with an academic health sciences librarian. Search terms included but were not limited to: “older persons, aged, elder,” “transition/transfer/move,” “emergency department/emergency room,” and “residential facility/long-term care/nursing home/assisted living.” A full search strategy is provided in Supplementary File 2. We searched five electronic databases in September 2022: EBSCOhost CINAHL Plus (1982 to present), Ovid Embase (1988 to present), Ovid MEDLINE (In-Process and other non-indexed citations; 1946 to present), Ovid PsycINFO (1987 to present), SCOPUS (1960 to present). We actively sought gray literature through searching Google Scholar, organizational and government websites, and requesting sources from health-system decision makers on our research team.

### Screening Procedures

Records were managed in EndNoteX9© to remove duplicate records. Unique records were exported to Rayyan (Ouzzani et al., 2016) to complete screening. Team members (KT, IA, JW, MD, FJ, GGC, GH, GVB, JF) independently screened 100 abstracts and met to finalize inclusion and exclusion criteria. Then, each abstract was reviewed independently by two of five team members (KT, IA, JW, SS, RD). We held consensus meetings between paired reviewers to resolve conflicts. If consensus could not be reached, a third team member (KT, TP) reviewed the abstract to make a final decision. We repeated these procedures for full-text screening.

### Data Extraction

We adapted a data extraction form, previously used by team members, to include concepts from the Care Transitions Framework (Dy et al., 2015). The extraction form was pretested among team members (KT, TP, IA, JW, MD, FJ, GGC, GH, GVB, JF), who independently extracted two articles. Data extraction elements included (a) study characteristics (e.g., author, year, country); (b) study design, theoretical framework, and sampling; (c) intervention characteristics (e.g., intervention type, targeted group, providers); (d) implementation processes and evaluation methods (e.g., descriptions of organizational characteristics influencing interventions, stakeholder engagement, fidelity, feasibility); (e) data analysis; (f) study results; (g) author descriptions of improved transitions from RACF to EDs, and (h) study limitations and author conclusions.

### Quality Assessments

We conducted quality assessments for all included studies using the Mixed-Methods Appraisal Tool (MMAT) version 2018 (Hong et al., 2018; Souto et al., 2015) but did not exclude studies based on quality. Team members (KT, TP, RD, SS, IA, JW) independently assessed each included study. We held consensus meetings to resolve conflicts.

The MMAT quality appraisal tool consists of two screening questions and five questions on methodological quality criteria for each type of research design. Each criterion has three response options: Yes (criterion met: score 1), No (criterion not met: score 0), Can’t tell (not enough information in the paper to judge if the criterion is met or not: score 0). We have provided individual study scores for each item, as is recommended, as well as individual study total scores in Supplementary File 2. We did not score all items of two studies based on lack of clarity of research question and relevance of data collected, as the remaining scores about study quality are based on positive or clear responses to these screening questions. Total quality scores for each study are provided in the study characteristics table (Table 2). Total quality score ranges from 20% (if only one criterion is met) to 100% (if all criteria are met). The study is considered of poor quality if it scores 20%, medium quality if it scores 40% or 60%, and high quality if it scores 80% or 100%.

**Table 2.** Characteristics of Included Studies

	Author(s), year, journal, country	Theoretical framework	Research design	Purpose	Sample and setting	Measurement tools for data collection, analysis, and outcomes	Descriptions of improved transition	Total quality score (%)
1.	Aizen et al., 2001 *Israel Medical Association Journal* Israel	Not reported	Retrospective cohort study	To evaluate the clinical effectiveness of a pilot project related to hospitalization of residential aged care facility (RACF) residents to an acute geriatric department	*n* = 126 nursing home residents admitted to the geriatric department: 80 directly from one of 11 RACFs and 46 residents through an emergency department (ED) in Haifa, Israel	Data collection: Clinical Dementia Rating Scale (CDR), and Katz Index of Activities of Daily Living. Admission records for other data Analyses: Descriptive analysis; group comparisons using student’s *t*-test to test intervention Outcomes: Length of stay, discharge disposition, mortality, cause of hospitalization, chronic medical condition, cognitive state, functional status at admission, and change of functional status during the hospital stay	To “…avoid some of the unwanted effects of emergency room transfer of nursing home residents without risking the patients’ health, and save [emergency room] resources” (p. 734; implicit in aim of program)	80
2.	Belfrage et al., 2009 *Medical Journal of Australia* Australia	Not reported	Not reported	To assess an RACF transfer-to-hospital envelope to support safe clinical handover from RACFs to the ED	*n* = 184 RACF staff, *n* = 30 ED staff, and *n* = 11 ambulance officers in 26 aged care homes, the EDs of six major metropolitan public teaching hospitals in Melbourne, and ambulance officers involved in transferring residents from RACF to hospitals	Data collection: Weekly data forms submitted by aged care homes Analyses: Descriptive analysis. Evaluation of intervention using written surveys, semi-structured face-to-face interviews, group interviews, discussion session, feedback and consultation Outcomes: Use, usefulness, and ease of use of the envelope; impact of using the envelope on clinical handover; awareness of the need for clinical handover; sustainability of the project	Not reported	80
3.	Burl et al., 1998 *Journal of the American Geriatrics Society* USA	Not reported	Retrospective cohort	To evaluate the impact of a geriatric nurse practitioner/physician team for enrollees of Medicare Health Maintenance Organization (HMO) in RACFs	*n* = 1,077 HMO residents residing in 45 proprietary and not-for-profit, licensed long-term care facilities in the HMO’s service area of central Massachusetts and in both skilled (Medicare-certified) and custodial beds: *n* = 663 residents cared for by physicians; and 414 cared for by geriatric nurse practitioner and physician teams	Data collection: Demographic, utilization, cost, and revenue data reviewed for the entire pool of residents for all services covered by HMO analyses: Cost–benefit analysis to test the impact of intervention Outcomes: hospital, ancillary services, skilled nursing facility and ED costs. Cost versus revenue for HMO enrollees (MD vs geriatric nurse practitioner [GNP]/MD) in long-term care	Not reported	80
4.	Cavalieri et al., 1993 *Journal of the American Osteopathic Association* United States	Not reported	Prospective cohort	To evaluate care provided by a geriatric assessment team based in the RACF	*n* = 69 consecutive long-term care patients randomly assigned on arrival to team (*n* = 33) and non-team (standard care, *n* = 36) conditions in the Health Care Center at Washington	Data collection: Medical chart review. Communication, ambulation, daily activities, excretion, and transfer (CADET) assessment for physical function, and Function, Reasoning, Orientation, Memory, Arithmetic, Judgment, and Emotion (FROMAJE) test for cognitive function Analyses: Unpaired *t*-test to test impact of intervention across groups Outcomes: numbers of consultations per service, frequency of hospital and ED visit, number of admission diagnoses, prescription medication, and mortality	Not reported	100
5.	Chan et al., 2018 *Internal Medicine Journal* Australia	Not reported	Not reported	To evaluate an acute geriatric outreach service on ED presentations from RACFs	*n* = 986 nursing home patients from 12 nursing homes presenting to any ED in New South Wales	Data collection: Administrative records of residents, database from the New South Wales ambulance service Analyses: Independent Student’s *t*-test to compare difference between the groups (pre- and postintervention), negative binomial regression to assess risk of ED presentation in each group (in post intervention period), and cost–benefit analysis to assess potential savings after intervention implementation Outcomes: ambulance transfer, ED presentation, potential savings after intervention	Not reported	60
6.	Conway, Higgins, et al., 2015 *International Emergency Nursing* Australia	Not reported	Pre–post-test study	To evaluate a nurse-led telephone support service, for RACF staff	Four RACFs located in a Local Health District in New South Wales, Australia: *n* = 50 staff from ED; and *n* = 26 staff from RACF	Data collection: Existing health service data, interview and focus group transcripts Analyses: Descriptive statistical analyses. Thematic analysis to assess the impact of intervention Outcomes: presentations to ED, triage category and diagnoses, ED length of stay (LOS), admissions to hospital and hospital LOS, impact on transfers, guidelines used in decision making	“… improved cognitive assessment, pain management, and care as older people transfer, in both directions between. RACFs and EDs” (p. 191; implicit during description of issue)	80
7.	Conway, Dilworth, et al., 2015 *Australian Health Review* Australia	Not reported	Case study	To describe an emergency service for aged care to enable point-of-care management of older adults residing in RACFs	Five EDs and 85 RACFs in the local health district, ambulance and primary and community care providers within a single Medicare Local in New South Wales, Australia	Data collection: focus groups, interviews Analyses: No specific analysis reported, but narrative summary of the impact of intervention Outcomes: ED presentations and admissions from RACFs, quality care focusing on needs of the older person	Not reported	80
8.	Dai et al., 2021 *Aging Medicine* Australia	Not reported	Interrupted times series	To evaluate an acute geriatric outreach service and its influence on hospital admissions from RACFs	The total number of beds in the 12 study RACFs was 1,421 in 2012. This number grew over time to 1,491 in 2019; over the 19 months of the subacute geriatric outreach service (SGOS) period (June 1, 2013 to December 31, 2014), there were 4746 geriatric department inpatient admissions, of which 812 (17.1%) were from a RACF. Over the 4 years of the acute geriatric outreach service (AGOS) period (2016–2019), there were 9,751 geriatric department inpatient admissions, of which 1,302 (13.4%) were from an RACF	Data collection: Extracted data from the acute geriatric outreach service database. Obtained admission data from hospital’s clinical information unit Analyses: Independent *t*-test to compare across time period to assess intervention impact on admission, negative binomial regression to assess risk of admission across the intervention periods Outcomes: Hospital admissions	Not reported	60
9.	Dalawari et al., 2011 *Geriatric Nursing* United States	A 2006 focus group study by Terrell and Miller developed a list of 16 important elements needed in the transition from nursing home-to-ED for patient care	Retrospective cohort study	To assess transfer form impact on communication between RACFs and EDs	*n* = 80 out of 306 patients aged > or = 65 years and transferred by Emergency Medical Services from nursing homes to Saint Louis University ED	Data collection: data obtained from patient electronic medical records Analyses: Descriptive statistics; Mann–Whitney *U* test to compare differences between two groups (intervention and no intervention group), Chi-square analysis, *t-*test Outcomes: availability of essential information needed for patient care, case resolution, and disposition status	“… relevant information being provided to emergency department staff” (p. 274; implicit in discussion)	100
10.	Hullick et al., 2016 *BMC Geriatrics* Australia	Not reported	Controlled pre–post study	To assess Aged Care Emergency services’ effect on RACF resident transfers to hospital	Four RACFs with a history of high ED presentations in a Local Health District in New South Wales, Australia: two facilities for the intervention group versus two facilities for the control group	Data collection: data extracted from electronic hospital admission records Analyses: Generalized estimating equations to estimate differential changes between intervention and control groups Outcomes: ED presentations, length of stay, hospital admission and 28-day readmission pre and post intervention	“[Facilitating] the opportunity to send patients home, rather than admit them to hospitals … through enhanced collaborative communication and decision making between ED and residential aged care facilities health care teams with a clearer understanding of the purpose of the transfer, what needed to be done by the ED, and the patient and their families’ wishes” (p. 7)	80
11.	Hullick et al., 2021 *Journal of the American Geriatrics Society* Australia	Not reported	Nonrandomized stepped wedge design	To evaluate the Aged Care Emergency program (ACE) and its impact on hospital admissions ED visits	9 EDs and 81 RACFs (RACF) across a large health district in New South Wales, Australia: *n* = 18,837 eligible hospital visits. For RACF residents, only hospital admissions routed through the ED were included in the study	Data collection: Administrative data Analyses: Descriptive statistics. Binomial mixed effects regression model to assess impact of intervention on the outcomes Outcomes: Hospital admissions, ED visits	Not reported	100
12.	Hullick et al., 2022 *BMC Geriatrics* Australia	Not reported	Pre–post, nonrandomized trial	To evaluate whether adding a video-telehealth consultation to the ACE program reduced hospital admissions and ED visits	Lake Macquarie, New South Wales, Australia, 13 RACFs referring patients to the same community hospital ED: of 13 RACF, 5 were intervention RACFs (used video-telehealth as part of the program) and 8 control Total sample: 1,271 (preintervention control: 461; postintervention control: 435; preintervention telehealth: 201; postintervention telehealth: 174)	Data collection: Hospital data Analyses: Descriptive statistics. Generalized linear mixed models (GLMM) to estimate differences in changes over time between control and intervention Outcomes: Hospital admissions, ED visits	Not reported	80
13.	Jensen et al., 2016 *Prehospital Emergency Care* Canada	Not reported	Retrospective cohort study	To evaluate an Extended Care Paramedic program and its impact on delivery in emergency care for RACF residents	Sample included all eligible calls attended by the Extended Care Paramedic program or emergency paramedics in Nova Scotia, Canada. The sample included 10 “Care by Design” long-term care (LTC) facilities. *N* = 360 LTC residents who had a 911 call made for them in the two study periods, *n* = 136 residents in the before period and *n* = 224 residents in the after period	Data collection: Records from emergency medical services, hospital, and LTC facilities Analyses: Statistical analysis, Chi-square tests, *t*-tests, and ANOVA to assess impact of intervention on outcomes Outcomes: number of EMS patients transported to ED and several clinical, safety, and system	Not reported	40
14.	Joseph et al., 2020 *Western Journal of Emergency Medicine: Integrating Emergency Care with Population Health* United States	Not reported	Retrospective, cohort study	To evaluate the impact of an RACF-based telehealth consultation service on hospital admissions, care escalation and financial implications of care	*n* = 4,606 residents who underwent an acute telehealth evaluation in 6 urban skilled nursing facilities in the Northeastern United States: *n* = 2,311 patients in the intervention group vs *n* = 2,295 patients in the control group	Data collection: Electronic health record data from telemedicine and ambulance transfers Analyses: Logistic regression to assess impact of intervention Outcomes: hospital admission, care escalation for conditions most amenable to on-site acute care	Not reported	60
15.	Kane et al., 2017 *JAMA Internal Medicine* United States	Not reported	Cluster- randomized clinical trial	To evaluate if supported implementation of the quality improvement program, Interventions to Reduce Acute Care Transfers [INTERACT] reduced hospital admissions and ED visits	*n* = 36,717 residents of 33 intervention and 52 control nursing homes: *n* = 9,050 and *n* = 8,380 residents in intervention nursing homes in the preintervention and intervention periods, respectively; and *n* = 14,428 and *n* = 13,472 residents in control nursing homes in the preintervention and intervention periods, respectively	Data collection: Minimum data set (MDS) to obtain resident data Analyses: Intent-to-treat analysis to compare the outcomes pre and post intervention Outcomes: hospital admissions, ED visits	Not reported	80
16.	Kwa et al., 2021 *Journal of the American Medical Directors Association* Australia	Not reported	Retrospective cohort	To examine RACF resident health service utilization when under different models of Residential in-Reach (RIR) services	*n* = 589 episodes of care (preprogram); *n* = 985 episodes of care (postprogram). Tertiary referral hospital in metropolitan Melbourne, with RIR catchment covering three local government areas, including 52 aged care homes	Data collection: Data obtained from clinical databases Analyses: Mann–Whitney *U* test and Pearson χ^2^ Outcomes: LOS, inpatient admission cost, ED attendances, hospital admissions	Not reported	80
17.	Ling et al., 2018 *Asian Journal of Gerontology and Geriatrics* Australia	Not reported	Not reported (retrospective cohort)	To examine an acute geriatric service and its impact on RACF residents’ ED presentation and hospitalizations	*n* = 276 residents (preprogram); *n* = 318 residents (postprogram) in the Bankstown-Lidcombe Hospital and nursing homes	Data collection: Medical records Analyses: Student’s *t*-test and Pearson χ^2^ to assess difference in outcomes during intervention and comparable prior months Outcomes: ED presentations, hospitalization	Not reported	100
18.	Lisk et al., 2012 *Archives of Gerontology and Geriatrics* United Kingdom	Not reported (operational framework reported)	Retrospective cohort study	To describe a health improvement cycle in RACFs to reduce ED visits from RACFs	Reviewed data of 1,954 residents admitted from nursing homes (NHs) for initial retrospective audit. Three nursing homes in the Surrey catchment area of Ashford and St. Peter’s NHS Trust with the highest number of multiple admissions were chosen for interventions to reduce admissions	Data collection: residents’ data from nursing homes Analyses: Chi-square to examine impact on outcomes compared between intervention period and nonintervention period. Outcomes: Hospital LOS, emergency hospital admissions	“Providing more bespoke care for this vulnerable patient group, keeping them out of hospital wherever possible” (p. 332; implicit in aim)	Not relevant
19.	Lloyd et al., 2019 *BMJ Quality Safety* United Kingdom	Enhanced Health in Care Homes (EHCH) framework	Subgroup analysis of a retrospective matched cohort study	To compare the effects and differences of hospital use between residents in residential care and nursing homes when employing an enhanced support program that included regular visits from named general practitioners and additional training for care home staff	People aged 65 years or older who moved to a participating residential care or nursing home from 2014 to 2016: *n* = 568. Thirteen resident care homes and 10 nursing homes were included from 6 local authorities in England	Data collection: Care home and administrative hospital data Analyses: Multivariable regression to assess outcomes in intervention and control groups; sensitivity analyses Outcomes: Potentially avoidable emergency admissions, unplanned emergency admissions, accident and emergency attendances, hospital bed days, hospital use, outpatient attendances, mortality	Not reported	100
20.	Madden et al., 1998 *Academic Emergency Medicine* United States	Not reported	Observational, cross- sectional, descriptive study	To describe the population transferred and reasons for transfer using transfer forms that were developed through a community-based task force assembled to address geriatric care To examine how transfer forms affected the relay of information and staff’s ability to ascertain important patient information	*n* = 344 patients transferred to the ED of the university teaching hospital that services a mix of insured and uninsured patients from seven nursing homes and two rest homes *n* = 34 nurses and *n* = 7 physicians in the ED	Data collection: Demographies and medical histories of patients from nursing home transfer form. Structured questionnaire completed by ED staff (nurses and physicians) Analyses: Descriptive analyses to describe the perception of staff on using the transfer form (intervention) Outcomes: Perspective on form use to improve transfer of information	“[Improving] transfer of information between institutional care providers” (implicit in conclusion)	60
21.	Marsden et al., 2018 *Australasian Journal on Ageing* Australia	Not reported	Descriptive, discussion study	To describe the Care coordination through ED, Residential Aged Care and Primary Health Collaboration (CEDRiC) project	*n* = not relevant; ED and Residential Aged Care and Primary Health in South East Queensland	Data collection: Observation, experience of implementation of intervention Analyses: Provides a narrative summary of how a care coordination-focused intervention project can decrease inappropriate hospital admissions Outcomes: Service coordination, inappropriate hospital admissions	An improved model of care was described as, “supporting the provision of additional clinical resources within RACFs; promotion of Advance Care Directives (ACDs) and End of Life Pathways for palliative care [7]; rapid older person assessment and CGA in the ED [8]; and enhanced education in gerontology care [9–12]” (p. 136)	Not relevant
22.	Mateos-Nozal et al., 2022 *Journal of the American Medical Director’s Association* Spain	Not reported	Not reported (feasibility study)	To examine the implementation of a hospital-based liaison geriatric unit	Geriatric department of a 780-bed public university hospital by recruiting three geriatricians and three advanced nurses specialized in geriatric nursing. This unit was shaped to proactively organize the care of all older persons living in the 31 NHs located in the hospital catchment area (approx. 600,000 persons, of whom more than 37,000 are older than 79 years)	Data collection: Online questionnaire Analyses: Descriptive statistics and narrative summary to describe impact of intervention on outcomes Outcomes: Hospital transfers, hospital admission, hospital use	“…improve[d] [nursing home] residents’ health care by direct patient care and by improving communication between the [nursing home] and the hospital through teamwork and telehealth” (p. 308; implicit in aim)	60
23.	McLane et al., 2022 *Canadian Journal on Aging* Canada	Not reported	Mixed- methods descriptive study design	To evaluate the use of a multi-setting documentation form across transitions and to assess healthcare professionals’ perspectives on the form’s feasibility, usefulness, and applicability	*n* = 244 eligible transitions occurred at one large urban teaching hospital ED and 11 LTC facilities that reported the highest overall numbers of transitions to the participating ED	Data collection: Chart review to obtain data on form use and form completion. Surveys to obtain data on practitioners’ perspectives Analyses: Descriptive statistics. Wilcoxon signed rank tests to compare perceptions of transition using form and not using the form. Inductive content analysis to assess participants’ concern about the form or transition process Outcomes: use of forms across transitions, rates of form and data element completion, feasibility of use, usefulness, and applicability of the form	“…to improve consistency of documentation and thus continuity of care by reducing information gaps at each step of the transition” (p. 6; implicit in aim)	100
24.	Shrapnel et al., 2019 *Aging Clinical and Experimental Research* Australia	Not reported	Pre–post study	To assess the Mater Aged Care in An Emergency (MACIAE) model and its effect on RACF resident outcomes	Sample included residents from Aged Care Facilities (ACFs) presenting at the ED of Mater Hospital Brisbane, Australia: *n* = 1,130 participants.	Data collection: Resident data obtained from Emergency Department Information Service database, i.e., Patient Manager database, and patient charts. Clinical Frailty Scale (CFS) to assess frailty. Risk of hospital readmission by the LACE index (which stands for Length of stay, Acuity of admission, Comorbidities, and recent Emergency department use), and cognition by the Mini-Mental State Examination Analyses: independent *t*-tests, and Chi-square tests to compare pre and post intervention findings. External cost–benefit analysis Outcomes: length of stay, ward admissions, 28-day representation rates	Not reported	80
25.	Stadler et al., 2019 *Journal of the American Medical Directors Association*United States	Not reported	Pre–post study	To assess the Reducing Avoidable Facility Transfers model on acute healthcare utilization	Postacute care and LTC residents of 3 rural skilled nursing facilities in collaboration with a geriatric practice in a tertiary academic medical center	Data collection: Transfer records from skilled nursing facilities. Questionnaire to assess acute care preferences at study end Analyses: Student’s *t*-tests to assess differences in outcomes pre and post intervention Outcomes: ED transfers and hospitalizations, advanced care planning status, hospital charges, standard MDS quality metrics	Not reported	80
26.	Terrell et al., 2005 *Academic Emergency Medicine* United States	Not reported	Pre–post study	To improve communication between extended care facilities (ECF) and the ED, using a one-page, standard ECF-to-ED transfer form with essential data elements	For the preintervention period, *n* = 130 cases transferred from 41 different ECFs to the ED For the postintervention period, *n* = 72 consecutive cases transferred from 10 ECFs to the ED	Data collection: ED patient charts, Analyses: Intent to treat analysis to assess effectiveness of intervention Outcomes: ECF transfers with successful documentation, total number of essential data elements documented and the total number of pages of information transported with each patient	Successful documentation of essential data provided to the ED with use of a one-page, standard ECF-to-ED transfer form	80
27.	Tsai and Tsai, 2017 *Journal of Clinical Nursing* Taiwan	Society for Academic Emergency Medicine (SAEM) Geriatric Task Force Framework	Feasibility study: survey development and Delphi method	To develop, validate, and test the feasibility of a checklist to facilitate the process of RACF to ED transition	*n* = 583 nursing home residents in Taiwan (705 transitions)	Data collection: Retrospective chart review for baseline data, telephone interview to obtain information on outcome variables, interviews with nurses Analyses: *t*-test to compare differences between pre and postintervention Outcomes: feasibility and benefit of using checklist, length of hospital stay, readmission	An improved transition was characterized by the continuity of medical information transferred across care settings	60
28.	Zamora et al., 2012 *Journal of Nursing Care Quality* United States	Not reported	Pre–post intervention study	To examine the implementation of a web-based referral system	Four NHs located with a 50-mile radius of the study institution: *n* = 313 patients transitioning from the NH to the ED	Data collection: Surveys to assess respondents’ perspectives. Administrative records for staff turnover, and frequency of web-based communications accompanying ED referrals Analyses: ANOVA and Tukey’s HSD test to compare pre- and postintervention implementation mean scores. Fisher’s exact test Outcomes: Compliance, adequacy of information, satisfaction with the information transmitted through the web-based referral system, costs of web-based referral system	Not reported	40

### Data Analysis

We employed numerical summary and qualitative content analysis to categorize study characteristics and substantive findings. Intervention categories were grouped by team members during data extraction based on similarities and differences in the author descriptions, nomenclature used, and the major intervention components. Findings were tabulated within these categories. We held team analysis meetings until we reached a consensus on the findings.

## Results

**Figure 1.**
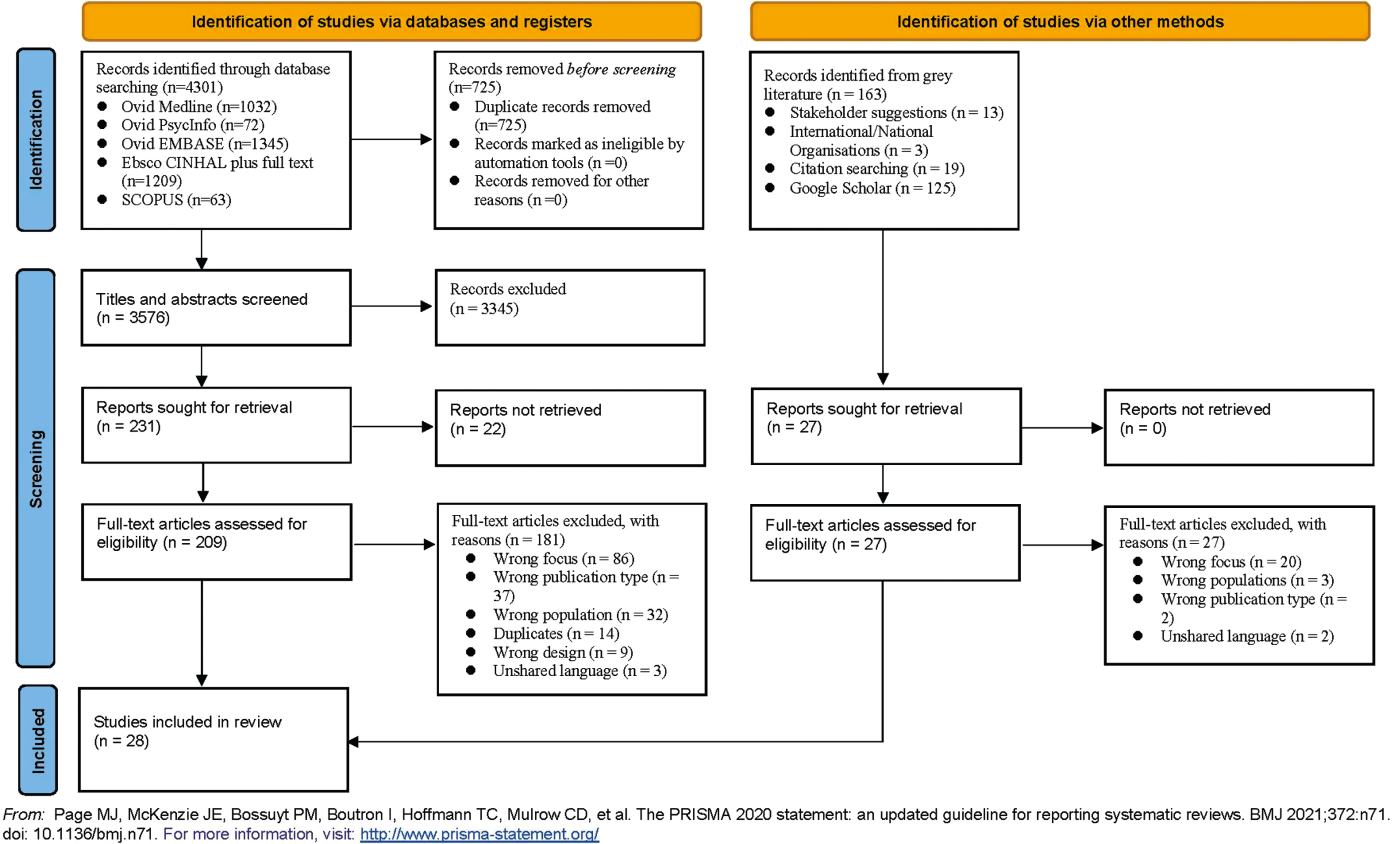
PRISMA-ScR diagram. PRISMA-ScR flow diagram (adapted from Page MJ, McKenzie JE, Bossuyt PM, Boutron I, Hoffmann TC, Mulrow CD, et al. The PRISMA 2020 statement: An updated guideline for reporting systematic reviews. BMJ 2021;372:n71. doi: 10.1136/bmj.n71. For more information, visit: https://www.prisma-statement.org/. PRISMA-ScR = Preferred Reporting Items for Systematic reviews and Meta-Analyses, extension for Scoping Reviews.

Once duplicates were removed, we screened 3,576 abstracts. Data were extracted from 28 articles after screening 209 full-text articles (see Figure 1).

### Study Characteristics

Studies were conducted in Australia (*n* = 12), the United States (*n* = 9), the United Kingdom (*n* = 2), Canada (*n* = 2), Spain (n = 1), Taiwan (*n* = 1), and Israel (*n* = 1). Of the 28 included studies, descriptions of improved transition were explicitly reported in 4 and were implicit either in the study’s aims, discussion, or conclusion in 7 (noted in the table as implicit), whereas in 17, they were not reported. Although our search criteria included a range of RACFs, no authors assessed care transitions solely from other congregate housing settings (e.g., assisted living). The most commonly reported study designs were retrospective cohort (*n* = 8), pre–post-test designs (*n* = 6), and prospective cohort (*n* = 2). Other designs were represented in only single studies (e.g., interrupted time series, nonrandomized step wedge, case study), and in four cases, no clear design was reported. Study characteristics are presented in Table 2.

Descriptions of improved transitions were not reported in 11 of 24 studies (Supplementary File 3). In 7 of these 11 studies, some understandings of improved definitions were suggested (labeled as “implicit” in Supplementary File 3) in the background or discussion of included papers. Only four studies included explicit descriptions of improved transitions (e.g., documentation of essential data provided to the ED with the use of a one-page, standard RACF-to-ED transfer form; Terrell et al., 2005).

### Quality Assessment Findings

Quality assessment scores (presented in Table 2 and Supplementary File 2) indicated that many studies included large, representative samples and had relatively high scores (80–100). Study weaknesses included incomplete and/or lower-quality data and a lack of adjustment for confounding factors.

### Study Findings by Intervention Categories

We identified the following intervention categories (Table 3): geriatric assessment teams or outreach services (*n* = 7), standardized checklists or documentation forms (*n* = 6), models of care to reduce or improve transitions from RACF to the ED (*n* = 6), telehealth services (*n* = 3), nurse-led care coordination programs (*n* = 2), use of geriatric acute-care departments to bypass traditional ED services (n = 2), an extended care paramedicine program (n = 1), and a web-based referral system (*n* = 1). Intervention characteristics and findings are presented in Table 2. Next, we highlight key findings across types of interventions.

**Table 3.** Included Interventions Categorized by Intervention Type

Author/year/country	Intervention type	Intervention implementation processes and measures	Contextual factors influencing intervention success	Substantive outcomes/results
Geriatric assessment or outreach services				
Burl, 1998 United States	Daily connections via telephone between geriatric nurse practitioner (GNP)/physician teams in long-term care, and their routine visits (routine visits alternated when needed and applicable)	GNPs mentored, assessed feasibility based on cost of intervention, and assessed utilization and cost to make group revenue neutral (managed by team vs MDs alone)	On site presence of practitioner/physician Understandings of legislative guidelines Reimbursement	***Substantive findings*:** Residents covered by the GNP/MD teams utilized fewer services, and total expenditure was significantly less for these residents (*p* = .011) ***Implementation findings*:** Significantly lower costs were found for ED (*p* = .001), hospital (*p* = .008), and skilled nursing (*p* = .001) for the GNP/MD. No differences in cost for “other” services, including ancillary services, lab, x-ray, drugs (*p* = .321)
Cavalieri et al., 1993 United States	Comprehensive Geriatric Assessment Team (CGAT) in nursing homes, which included geriatricians and GNPs	Not reported	Not reported	***Substantive findings*:** No significant difference in longevity (274 vs 235 mean days alive since admission, but nonsignificant), hospital admissions, or emergency room visits when compared between CGAT and non-CGAT service ***Implementation findings*:** Not reported
Chan et al., 2018 Australia	Acute geriatric outreach service (AGOS) to be accessed at nursing homes (Monday to Friday, 9 am to 5 pm) for residents who experience certain conditions/symptoms (e.g., fever ≥38 Celsius, cellulitis, heart failure)	Not reported	Difference in staffing or expertise in nursing homes Ability to provide rapid response Support, training and skills for nursing home staff Recognition of service/intervention Seasonal variations in ED presentations	***Substantive findings*:** The cost–benefit ratio of the intervention was 5.18, indicating that for every $1 spent on AGOS, a saving of $5.18 was realized from reduced admission and ambulance transfer frequency ***Implementation findings*:** Safe and sustainable intervention as there was only one case of treatment complication in 2 years and no unexpected mortality
Dai et al., 2021 Australia	Geriatric outreach service in which geriatricians and nurses triage referrals from nursing home to assess and manage patients. Treating in place is sought, if possible	Not reported	Subacute geriatric outreach service implementation Case mix (top conditions were respiratory infections, urinary infections, dehydration)	***Substantive findings*:** 15.7 fewer monthly admissions when compared to previous service. Risk of admission reduced by 36.1% (incidence rate ratio = 0.64; 95% CI: 0.58–0.71; *p* < .001), adjusted for seasonality ***Implementation findings*:** Not reported
Hullick et al., 2016 Australia	Aged care emergency (ACE) service to support enhanced collaborative communication and decision-making between ED and residential aged care facilities healthcare teams	Two-hour education program to clinical staff was provided to support a manual of algorithms to use	Leadership Engagement of direct care staff Culture of quality improvement	***Substantive findings*:** Significantly higher odds (59%) of hospital admission among intervention RACF groups vs control groups across both pre- and postintervention periods (*p* = .0002). The decrease in length of stay was greater in intervention group, although not statistically significantly (*p* = .18) ***Implementation findings*:** Not reported
Hullick et al., 2021 Australia	ACE program, nurse-led service, to manage acutely unwell residents. It includes telephone support, evidence-based algorithms, defining goals of care for ED transfer, case management in the ED, and an education program	Specialist registered nurse (RN) (with administrative support, and help of nurse educator) undertook comprehensive geriatric assessment, organized referrals, advocated for older people and educated ED staff, and provided ACE telephone consultation. Upon arrival, residents were case managed, discharged home wherever reasonable	Telephone consultation service for RACF staff Evidence-based algorithms for common symptoms Proactive case management by specialist aged care nurses Education in communication techniques for nurses Collaboration across RACFs, GPS, ambulance, local hospitals and EDs Identification of barriers and facilitators Ongoing change management and coordination for ACE key stakeholders	***Substantive findings*:** Lower 7-day ED representations (5.7% vs 4.9%). 30-day readmissions fell from 12% for the control period to 10% for the intervention period. Higher proportion of ATS triage two patients in postintervention (15% vs 12%). Admissions to critical care wards lower (2.9% vs 4.2%) ***Implementation findings*:** Not reported
Ling et al., 2018 Australia	AGOS	Not reported	Operating hours of program Newness and awareness of program Geriatricians being the only point of contact Presence of triage system or referral pathway Seasonal variation in presentations to ED	***Substantive findings*:** Increased [ED] discharge rate and decreased hospital admission rate for nursing home residents who presented with fall without fracture, respiratory, gastrointestinal, or cardiovascular complaints ***Implementation findings*:** Not reported
Standardized documentation forms				
Belfrage et al., 2009 Australia	Transition checklists (from NH to ED) to ensure medical information continuity	Evaluation of envelope usefulness (feedback on layout, design, content, and how to support ongoing national use) via written survey, group interviews, targeted interviews with aged care home and ED staff, as well as ambulance officers and feedback/consultation with advisory management and reference groups	Low cost Familiarity with tool Clear and simple instructions for tool use Minimal support and training requirements Key challenge for ongoing use of envelope: Supply and distribution issues	***Substantive findings*:** Implementation findings are substantive findings in this study ***Implementation findings*:** Discrepancy in reporting envelope use (care home data indicates 89% use, and ED data indicates 84% use). 99% reported it was useful and 90% reported it was easy to use. ED staff highlighted envelope was useful for background knowledge of aged care sector and discharge planning, while ambulance officers pointed that the handover information was more organized and most interviewees (around 80%) believed it improved handovers too. Around 90% reported that they would continue to use the envelope in all resident transfers, and recommend its use in another setting. All interviewees reported the envelope increased awareness about clinical handover importance
Dalawari et al., 2011 United States	Transfer documentation form (transition reasons, medical and sociodemographic information, advanced directives and contact information)	Faculty meetings among ED physicians were held to rate the relative importance of each item and ED nurse approval of items were obtained	Time limitations in emergency situations Frequent NH staff turnover Complexity of transfer form layout and design Not having the information available to complete the transfer form	***Substantive findings*:** No differences in case resolution time, admission, or discharge status ***Implementation findings*:** Transfer forms more likely to be completed in the afternoon hours as compared to overnight hours (2300–0659)
Madden et al., 1998 United States	Standardized transition documentation form	Forms pilot tested for 2 months: forms (on bright pink paper) from nursing homes , and forms from the ED (on bright blue paper). Education on use of forms for emergency medical services personnel, feedback obtained, and pink form used for this study	Partnerships with and existence of community coalition Inclusion of providers early on in the process lead to enhance understanding of and willingness to address the issue	***Substantive findings*:** Nurses and physicians using the forms reported that patient care and understanding was a lot easier most of the time (around 95% of the time). Around 88% of the time it was easier for them to understand the list of medications the patient was on. 93% of the ED staff reported taking <5 min to gather information for patients with the forms and 56% reported without the form, it took them >10 min ***Implementation findings*:** Hospital ED staff, nursing homes, and EMS personnel wanted to continue using it even after the study completion
McLane et al., 2022 Canada	Standardized transition documentation form to be used across settings	Instruction manual (both paper and electronic), and in-person one-hour training (for night and day staff) provided to staff in long-term care, emergency medical services and the ED. Research staff were available for follow-up questions and additional training was offered	Values and perspectives on formal health record keeping Existence and use of other documentation forms Trust/distrust of information provided by other healthcare professionals Time pressures Lack of single point of leadership and oversight for entire transition process	***Substantive findings*:** Implementation findings are substantive findings in this study ***Implementation findings*:** Most ED participants reported that ease of identifying resident information was improved with form use (significant at <.05) except for those related to current medication list, medical history and laboratory tests/x-rays. Long-term care (LTC) survey results on ease of use were nonsignificant. Some participants reported the form as stressful, redundant, time-consuming to complete, and being frustrated at sharing documentation across care settings, whereas some reported that it was easy to use and was comprehensive. Some participants prioritized form completion over direct patient care. Median uptake of forms was 43%. Only 1 form of 100 was completed by all care settings. Of 74 forms in which both pages were accounted for, all LTC portions were completed, 26% of EMS portions were completed and 7% of ED portions were completed
Terrell et al., 2005 United States	One-page standardized transition documentation form to be used across settings	Proportion of transfers with successful documentation, which was defined as documentation of 9 or more of 11 essential data elements, total number of data elements, and the number of pages transported with the resident	Not reported	***Substantive findings*:** Proportion of transfers with successful documentation significantly increased by 19.3% (95% CI = 4.0% to 34.7%); the number of essential data elements significantly increased by 0.8 elements (95% CI = 0.3–1.4 elements) ***Implementation findings*:** Not reported
Tsai and Tsai, 2017 Taiwan	Checklist for transitions (from NH to ED) to ensure medical information continuity	Interviews with NH nurses for initial items. Obtained stakeholders’ feedback using Delphi method. Feasibility assessed using baseline data collection for 6 months	Checklist could easily be transferred to electronic application Difference in staffing (nurse practitioners versus other nursing designations)	***Substantive findings*:** No significant differences in length of hospital stay and 30-day readmission rate. No findings on continuity of medical information reported ***Implementation findings*:** Length of hospital stay and 30-day readmissions rate reported as measure of feasibility and benefit of checklist use. No significant differences reported
Models or programs of care				
Conway, Dilworth, et al., 2015 Australia	Multiorganization ACE service designed to enable point-of-care assessment and management	ACE manual was provided, one point-of-contact telephone consultation, education on intervention provided, explicit resident goals of care, proactive case management within the ED, change management coordination, and maintenance of collaborative relationships	Provision of training and resources available health care assistants (HCAs) access to RNs when needed Consistent clinical handover Funding model (mixed in this case) Acknowledgment of stakeholders’ organizational funding structures, reporting and business rules Regular interagency meetings Presentations at regional, state and national levels Stakeholder engagement and consultation Consultation with relevant medical specialists Online resources linked to the local health pathways program for GP referrals	***Substantive findings*:** Implementation findings are substantive findings in this study ***Implementation findings*:** Residential aged care facility (RACF) staff reported feeling equipped, supported and empowered to care for residents**.** GPs reported better systematic management, improved communication among RACFs, GPs, and emergency departments (EDs). Positive feedback on education sessions
Kane et al., 2017 United States	INTERACT program—suite of tools based on three strategies that focused on recognition, evaluation, and management of acute conditions, supportive tools (e.g., communication, documentation, decision) for on-site (nursing home [NH]) effective management, and emphasis of advance care planning, hospice, and palliative care as an alternative to hospitalization	Prior quality improvement program-based strategies employed, evaluation and training facilitated by champions and co-champions, and root cause analyses on transitions using the INTERACT tool were submitted, evaluated and used to discuss study progress and ways to overcome challenges in implementation	Rural location Number of Medicare-certified beds For-profit status Number of certified nursing assistant (NAs), Licensed Practical Nurses (LPNs), RNs hours per resident day reported at baseline (in 2012) Occupancy rate Percent long-stay residents/resident condition Quality performance on NursingHomeCompare Nature of training and support provided Quality of staff Concerns over legal and regulatory liability Motivation to reduce hospitalizations/ED visits and readmissions based on influence of value-based care initiatives	***Substantive findings*:** No significant differences in ED visits resulting in hospital admissions ***Implementation findings*:** Participants attended 67% of online webinars and completed 52% of online course modules. Intervention sites submitted 63% of root cause transitions requested and 52% of monthly support/or feedback calls. Commonly cited barriers included scarce resources, staff resistance, competing demands, and instability of NH leadership; commonly cited facilitators included organization-wide involvement, persistence and oversight, adequate training, and leadership support
Kwa et al., 2021 Australia	New model of care-service redesign that included suite of services (e.g., access to diagnostics, IV/IM or palliative care medications, hydration) provided by a nurse and consultant physician during weekdays and provision of referrals by staff at RACF, or hospital	Changes in services (e.g., geriatrician in the ED, liaison nurse role integration, stakeholder engagement) were made to improve quality of care	Staffing (change from rotating physicians for medical support to single consult leading the program) Physician practices Proactive stakeholder engagement Increase in catchment area	***Substantive findings*:** For those ED presentations that required hospital admission, median length of stay (LOS) [2 (IQR 1–6) days vs 2 (IQR 1–5), *p* = .98], median cost per inpatient admission [$3,712 vs $3,787 ($USD 2,570 vs $2,622), *p* = .27] were not significantly different between the 2 periods. Inpatient care cost per 100 RACF beds was lower in period 2 [$145,607 vs $117,531 ($USD 100,829 vs $81,383), *p* < .001] ***Implementation findings*:** Not reported on
Lisk et al., 2012 United Kingdom	Includes suites of services such as medical advisory meetings with GP, daily telephone advice, medi-home, end-of-life care, and email alert system	To reduce admission, need for medical input for residents was discussed between primary doctor and care home manager, geriatric consultants provided telephone advice, specific nursing care for those awaiting discharge with no need of acute services, and end-of-life care documentation to facilitate expression of residents and caregivers wishes. Email alert system for transitions, and geriatrician review before discharge and liaison with admitting team and primary doctor were used as approaches to reduce the LOS.	Need or capacity for training in intravenous therapy in NH Education of managers Actively engaging NH managers and staff Use of locum GPs on weekends	***Substantive findings*:** Reduction of 57 bed days for length of hospital stay over a 3-month period for intervention RACFs (90 to 33 days) ***Implementation findings*:** Positive reception among GPs
Lloyd et al., 2019 United Kingdom	Enhanced support program that included regular visits from named general practitioners and additional training for care home staff	Not reported	Differences in how the community nurse support and training operated in facilities Resident medical complexity Access to 24 hours’ access to nursing home expertise Usual care practices	***Substantive findings*:** The adjusted rate ratio associated with receiving the enhanced support was 0.50 (95% CI 0.30–0.82), equivalent to on average 0.20 less of these admissions per person per year among the residents (95% CI−0.28 to −0.07). Lower rates of emergency admissions than matched controls (0.59 vs 0.93 per person per year, adjusted rate ratio 0.60, 95% CI 0.42 to 0.86), equivalent to 0.37 less emergency admissions per person per year (95% CI −0.54 to −0.13) ***Implementation findings***: Not reported
Stadler et al., 2019 United States	Facility transfers model that includes team that manage longitudinal care and after-hours call, elicitation of advance care plans and preferences regarding acute care, standardized communication process, and a biweekly case review of all ED transfers	Five physicians, three NPs, and one physician’s assistant managed all care and after hour call (2–3 in each building and one on site every weekdays). Advance care plans and Provider Orders for Life Sustaining Treatment (POLST) forms as standard practice and 24/7 electronic access to it, and reviewed monthly. Education for nursing staff, and bimonthly meetings to discuss 15–20 cases. Encouraged to prioritize needs and directly engage with patients	Integration of intervention as part of standard practice Systematic goals of care discussions Meeting after facility admissions and early and ongoing conversations to identify those at high risk Clinician familiarity with care of frail older adults and facilities Active and early engagement of on-call physicians Physicians talking directly to families Regular review of all ED transitions	*S* ***ubstantive findings*:** Monthly LTC hospitalizations significantly decreased by 62.4% postintervention ***Implementation findings*:** Not reported
Telehealth services				
Conway, Dilworth, et al., 2015 Australia	Nurse-led telephone support service (from 0800 to 2000 hr, till 4:30 on weekends and holidays), clinical guidelines (adapted with consultation with RACF staff) and education for RACF staff	Health data were used to determine implementation issues pertaining to guidelines used by RACFs and the ACE APN in decision making. Interviews or focus groups included staff from the ED (ACE APN, ED RNs, nurse managers and Emergency Physicians), RACF staff and primary care medical providers (no response from this last group). Emergency medical services staff provided anecdotal feedback	Lack of primary care medical providers in RACF Staff mix Scope of practice of staff within RACF RACF policies and procedures On-site education to staff and practice Training, guidelines, support and advice from the APN when dealing with common emergencies enhanced staffs’ knowledge and skills	***Substantive findings*:** 19% decrease in ED admissions**,** and 35% decrease in total inpatient days ***Implementation findings*:** RACF staff reported becoming more confident in their ability to manage people in the facility respondents. However, others expressed concern that following advice to keep residents in the RACF to monitor them may result in the person’s condition worsening before being transitioned
Joseph et al., 2020 United States	Telehealth consultation service staffed by emergency physicians	Intervention group were provided on-demand consultation by an EP (facilitated by a clinical care specialist [CCS] who uses a cart with point-of-care labs, electrocardiograms, telemetry, and ultrasound), acute care services, order sets and pathways used to streamline transfer decisions. Residents in control group were transferred via ambulance to the ED of an urban tertiary care hospital with 55,000 visits annually	COVID-19 pandemic Ability of clinical care specialist to fulfill medication orders and re-initiate consultation Expanded diagnostic tools, allowing the Emergency Physicians (EP) to conduct much of an ED workup in situ	***Substantive findings*:** Care escalation (hospital admission vs ED transfer) by resident condition: Congestive heart failure (OR 0.08 [0.06–0.11] < 0.001), chronic obstructive pulmonary disease (OR 0.13 [0.10–0.18]< 0.001), and diabetes mellitus (OR 0.21 [0.15–0.29] < 0.001) ***Implementation findings*:** Not reported
Hullick et al., 2022 Australia	Video-tele health consultation to the established ACE program	Video-telehealth pathway activated for hospital transfer (7 days a week, 8 am–4 pm), and real-time interactive communication between the resident (RACF staff support provided) and ED. For a new or returning patient to the RACF, a planned video-telehealth enhanced clinical handover occurred. Portable computer tablet in RACF, and computer on wheels in hospital was used when resident was unwell	Established hospital RACF avoidance program Financial incentives Staff valued telehealth handover Relationship and collaboration	***Substantive findings***: No significant differences in hospital admission or ED visits after the introduction of video-telehealth (IRR = 0.98, 95% CI 0.55–1.77 and 0.89, 95% CI 0.53–1.47), respectively ***Implementation findings*:** Not reported
Nurse-led care coordination				
Marsden et al., 2018 Australia	Nurse-led multidisciplinary care coordination through the Emergency Department, Residential Aged Care and Primary Health Collaboration (CEDRiC) project.	Two Geriatric Emergency Department Intervention (GEDI) clinical nurses rostered to the ED, provided a clear communication pathway (facilitated the selection of most appropriate one) for transfers, focused on shared decision making (nurses, families, patient, and carers, targeted geriatric assessment for high-risk older adults). Additional consultation (e.g., emergency physician geriatrician) included if needed. Health Intervention program for seniors using nurse practitioner candidate (NPC)	Commitment of key staff Staff mix of care team Nurse practitioner ability to prescribe medication or bill for service Funding and supports available	***Substantive findings*:** Intervention streamlined transfers, subsequent assessment and admission to the ED; enhanced knowledge and skills of RACF staff, and enabled the identification of resident deterioration ***Implementation findings*:** Communication strategies were adopted after challenges addressed, these included development of brochures, staff education sessions, utilization of high visibility bright pink shirts, and a GEDI communication board. In the RACF, staff familiarization meetings with the NPC, staff education, and ensuring the NPC supplemented primary carers
Shrapnel et al., 2019 Australia	Nurse-led, person-centered model of care for older adults	Clinical liaison and telephone support for ACF/GPs for clinical decision-making (deteriorating patient, future care), hospital-wide care coordination (early identification, single point of contact, communication), shared accountability for care, ACP support (initiation, review, completion), and GP self-management education support for patients	Use of existing care services in the community Age of residents in RACF presenting to the ED Resources and capacity building Buy-in from stakeholders	***Substantive findings*:** Reduction in ward admissions by 31.3%, reduction in ED presentation by 13.2%, reduction in End-of-Life in-patient episodes by 88%. Cost–benefit was 10:1, which demonstrated the cost effectiveness of the intervention ***Implementation findings*:** Not reported
Use of an acute-care geriatric department				
Aizen et al., 2001 Israel	Use of an acute-care department of geriatric hospital to bypass traditional ED	Not reported	Not reported	***Substantive findings*:** Significantly higher prevalence for age, febrile disease, chronic obstructive pulmonary disease (COPD) on admission, heart disease, hypertension, dementia and gastrointestinal disease. Functional status of patients did not change from admission to discharge for 80.4% of ED patients and 81.2% of direct geriatric hospital admissions. Functional status improved for remainder of patients ***Implementation findings*:** Not reported
Mateos-Nozal et al., 2022 Spain	A hospital-based liaison geriatric unit with geriatricians and advanced nurses who specialized in geriatric nursing	Visited RACFs to explore first COVID-19 issues, introduced the unit to the teams and identified key stakeholders. Online quarterly census of residents’ characteristics sent to ED and hospital specialists. Alerts added to resident records. Consultation with the geriatric unit if needed, and communication with RACFs was protocolized. Template used for resident information before outpatient visits. Co-managed telehealth programs and drug protocols for follow-up plan Epidemiology support during COVID-19 waves	High vaccination rates Proactive assessments using electronic alerts Liaison unit within hospital Physician and nurse availability at least one shift a day	***Substantive findings*:** Coordinated care occurred with specialists in 67% of assessments**,** treatment changed in 33% of assessments**,** hospital admissions prevented for 20% of assessments**.** Of the 90 assessments in hospital, 43% aimed at improving coordination between acute care and RACF, 36% were to support direct patient care and 11% to manage geriatric syndromes. 66% of hospital discharges were planned through scheduled follow-up calls from unit to RACF staff. ***Implementation findings*:** Global satisfaction with intervention was 4.7/5 (*n* = 44)**,** improved accessibility to hospital and telehealth resources was 4.7, easier health coordination was 4.6, hospital drug availability was 4.6, co-management with specialists was 4.5**, and** improved COVID-19 support was 4.4**.** RACF staff reported agility, decision-making support and decreased numbers of ED referrals and hospitalizations from unit
Extended care paramedic program				
Jensen et al., 2016 Canada	Extended care paramedic program (ECP)	Not reported	Time it took to develop a care plan, consult with physicians and the ED, deliver treatment, and communicate with staff and families	***Substantive findings*:** The overall number of patients who were admitted to the hospital was not different between study periods. In the after period, fewer patients who received care from ECP were admitted compared to those from emergency paramedics. The response and scene times were statistically significantly longer when ECP was involved in the call compared to emergency paramedics in the after phase. Differences were not observed in the length of time EMS crews spent in the EDs in the before or after periods, or in the subgroup analysis of the after period ***Implementation findings*:** Not reported
Web-based referral system				
Zamora et al., 2012 United States	A web-based, password-protected referral page with required information fields related to patient information, clinician information (the clinician filling out the form) and bed-holding policy information. The completed form ports automatically to the patient electronic health record and can be accessed externally by clinicians with permissions through accepted passwords	Presentations and good communication with leadership, nursing staff training sessions and reference manuals provided, weekly to biweekly visits from research group, option to print the form provided, meetings with the ED house staff and various forms of reminders through the ED program director, and physician satisfaction surveys	Presence of other competing platforms Lack of availability and/or access to computers for nursing facility staff Availability and input of corporate technical support Outdated certificates of authenticity provided by the web programs that required the construction of a unique program for the online referral page Staff computer literacy Electronic medical record Staff and administrative turnover Pay and technological-savvy staff and administration	***Substantive findings were implementation findings***. ***Implementation findings*:** Compliance was 22.7% across included facilities. ED physicians reported being “completely satisfied” 52% of the time (*n* = 13) in regard to the overall information transmitted through the web-based referral system. ED physicians reported that 8.47 versus 7.22 previously, of 9 possible categories of information on the electronic referral had adequate information (*p* < .003)

*Notes*: Types of interventions to improve transitions from facility-based care settings to the ED included: geriatric assessment teams or outreach services (*n* = 7), standardized checklists or documentation forms (*n* = 6), models of care or suites of tools to reduce or improve transitions from RACF to the ED (*n* = 6), telehealth services (*n* = 3), a nurse-led care coordination program (*n* = 2), use of an acute-care department in a geriatric hospital to bypass traditional ED services (*n* = 2), an extended care paramedicine program (*n* = 1), web-based referral system (*n* = 1). CI = confidence interval; ED = emergency department; IQR = interquartile range; OR = odds ratio.

#### Geriatric assessment teams or outreach services

Seven studies examined how geriatric-focused assessment teams or outreach services resulted in improved and/or reduced long-term care (LTC)–ED transitions (Table 3). These teams were comprised primarily of geriatricians and specialized nurse practitioners, and to a lesser extent other allied health professionals. Although most interventions aimed to primarily reduce these transitions, the authors also examined the improved outcomes for transitions that did occur. The authors reported mixed results for interventions involving geriatric assessment teams or outreach services. Results ranged from significantly decreased hospital admission rates (Ling et al., 2018), decreased odds of hospital readmissions (Hullick et al., 2021), increased odds of hospital admissions (Hullick et al., 2016), and no significant effect on hospital admissions (Cavalieri et al., 1993). Two studies reported cost savings by using fewer services, consults, and the use of skilled nursing staff rather than a physician-only approach (Burl et al., 1998; Chan et al., 2018). The authors of one study presented cost savings as evidence of implementation effectiveness (Burl et al., 1998). The authors of another study concluded that their intervention was safe and sustainable based on only one treatment complication event in 2 years (Chan et al., 2018).

Although no authors discussed if or how they explicitly and proactively contextualized the intervention they examined, several (*n* = 6) discussed contextual factors that may have influenced study results. Examples include seasonal variation in ED transfers (Chan et al., 2018; Ling et al., 2018); different understandings of RACF legislative guidelines (Chhabra et al., 2012); differences in staff mix, availability, and expertise (Burl et al., 1998; Chan et al., 2018); the ability to provide rapid responses in RACF (Chan et al., 2018); availability of ambulance services, awareness of geriatric assessment teams or services (Chan et al., 2018; Ling et al., 2018); and factors related to leadership, a culture that supports quality improvement and a community of practice (Hullick et al., 2016, 2021).

#### Standardized checklists or documentation forms

Authors in six studies assessed how standardized documentation forms improved RACF to ED transfers (see Table 3). Two of these studies reported no statistically significant findings related to case resolution time, admission and discharge status, length of hospital stays, and 30-day readmission rates (Dalawari et al., 2011; Tsai & Tsai, 2017). The authors of a third study reported that healthcare professionals felt that the form was useful and improved handover processes, and was also a sustainable strategy to improve care transitions (Belfrage et al., 2009).

The authors of three studies evaluated healthcare professionals’ perspectives on whether using standardized documentation forms improved their understanding of patient needs and ability to care for patients (Madden et al., 1998), and also assessed form uptake (Belfrage et al., 2009; McLane et al., 2022) and item completion (McLane et al., 2022; Terrell et al., 2005). Belfrage et al. (2009) reported that form uptake was high (89%) for eligible cases, whereas McLane et al. (2022) reported lower form uptake (43%; McLane et al., 2022). Item completion improved or was perceived to have improved (by RACF, emergency medical services [EMS], and ED staff) in both studies (McLane et al., 2022; Terrell et al., 2005).

Generally, the authors did not explicitly describe how they embedded the form content and documentation process to meet the needs of study participants (as in these studies, the authors led form development or adaptation). McLane et al. (2022) reported that form content was based on findings from earlier research identifying issues that residents, family members, and staff reported during the transition process. The authors discuss how contextual factors influenced intervention implementation and success. Key factors included early engagement with relevant stakeholders (Madden et al., 1998); complexity and layout of the form (Dalawari et al., 2011); recognizing that participants had limited time in emergency situations (Dalawari et al., 2011; McLane et al., 2022); and ensuring that processes aligned with healthcare professional values about formal record keeping (McLane et al., 2022). The noted challenges included the use of multiple (existing and new) forms (McLane et al., 2022), lack of a single point of leadership and oversight during a transition, and staff mix and turnover (Dalawari et al., 2011; Tsai & Tsai, 2017).

#### Models or programs of care

Five studies examined multiple interventions presented as a model of care developed to improve RACF to ED transitions. These approaches included various services (e.g., medical advisory meetings, after-hours telehealth, homecare services; Lisk et al., 2012), tools (e.g., INTERACT, a quality improvement program that includes educational and clinical tools to improve care for residents in RACFs; Pathway Health, 2023), and improved access to healthcare professionals to support the assessment and overall management of care for RACF residents.

Findings from this type of intervention were largely positive. Monthly hospitalizations significantly decreased by 62.4% postintervention in one study (Stadler et al., 2019) and ED admissions were significantly lower in another (Lisk et al., 2012; Lloyd et al., 2019). Lisk et al. (2012) found that their suite of services significantly reduced the length of hospital stay. The authors examining the INTERACT program (Kane et al., 2017) found no significant reductions in hospitalizations. The authors from three studies reported that participants provided positive feedback about the intervention (Conway et al., 2015; Kane et al., 2017; Lisk et al., 2012).

Examples of how contextual factors influenced their study results include funding models and how this influenced researchers’ ability to provide training and additional resources (Conway et al., 2015; Lisk et al., 2012; Lloyd et al., 2019), and benefits of integrating the intervention into standard and/or usual care practices (Lloyd et al., 2019; Stadler et al., 2019). Kane et al. (2017) posit that intervention effectiveness was influenced by facility for-profit status, quality performance standings on national websites, and staff motivation to reduce hospitalizations and readmissions based on value-based care initiatives (Kane et al., 2017).

#### Other intervention types

Other interventions used to improve RACF to ED transitions included telehealth services (Conway et al., 2015; Hullick et al., 2022; Joseph et al., 2020), nurse-led care coordination interventions (Marsden et al., 2018; Shrapnel et al., 2019), the use of a geriatric acute care department (Aizen et al., 2001), an extended care paramedicine program (Jensen et al., 2016), and a web-based referral system (Zamora et al., 2012).

##### Telehealth services

Results pertaining to telehealth services were mixed. The authors in one study reported significantly lower odds of hospital admissions (Joseph et al., 2020). Hullick et al. (2022) reported no significant reduction in admissions using telehealth. Conway et al. (2015) reported that the use of telehealth decreased total inpatient hospital days and concluded that this intervention gave RACF staff more confidence in their ability to manage resident care (Conway, Higgins, et al., 2015). The authors reported that contextual factors influencing intervention implementation and success included the coronavirus disease 2019 (COVID-19) pandemic (Joseph et al., 2020), differences in staff mix and scope of practice, RACF policies, lack of primary care medical providers on site, and limited ability of specialists to fulfill medication orders and re-initiate consultation (Conway et al., 2015; Joseph et al., 2020).

##### Nurse-led care coordination

The authors in two studies investigating nurse-led care coordination interventions reported reductions in ward admissions and end-of-life inpatient episodes (Marsden et al., 2018; Shrapnel et al., 2019), and enhanced cost effectiveness in one study (Shrapnel et al., 2019). Marsden et al. (2018) adapted their intervention based on participant feedback, specifically by developing brochures and a communication board, conducting staff education sessions, and providing bright t-shirts for care team members (Marsden et al., 2018). The authors discussed key contextual factors influencing intervention effectiveness and implementation, including supports provided for the intervention, the commitment of key staff, staff mix and nurse practitioner ability to practice to full scope, the use of existing care services, buy-in from stakeholders, and age of the residents presenting to the ED (Marsden et al., 2018; Shrapnel et al., 2019).

Generally, remaining interventions (i.e., extended paramedicine program, web-based referral system, acute geriatric departments) reduced the number of residents transferred to acute care, but did not impact other outcomes such as the extent or duration of functional decline during hospital stay (Aizen et al., 2001). In their assessment of the extended care paramedicine program, Jensen et al. (2016) reported no significant differences in EMS response and scene time, ED length of stay, and hospital admission and relapse. Nonsignificant increases in scene times were reported to be influenced by the time it took paramedics to develop a care plan and to consult with the physician/ED while concurrently communicating with families. Most ED physicians were completely satisfied with a web-based referral system to help guide RACF to ED transitions (Zamora et al., 2012). The authors discussed the importance of technical support, access to computers, and staff computer literacy to ensure successful intervention implementation.

## Discussion and Implications

Our study augments existing systematic reviews by (a) expanding on the breadth of research designs examined, (b) employing a theoretical framework, and (c) demonstrating the (limited) extent to which the researchers currently have tailored their interventions to care settings and/or assessed implementation effectiveness. This research is unique and advances knowledge by focusing on strategies that have been used to improve transitions from RACF to EDs. From our examination and critique of how improvement is described in the literature, we illustrate the need for researchers to clearly articulate the concept of “improvement”; to explain in greater detail how interventions designed to achieve this goal are developed and contextualized to align with care environments; and to create more fulsome evaluation platforms that assess the concepts of intervention and implementation effectiveness. In doing so, we can differentiate promising practices that suffer from inadequate implementation strategies from interventions that are less likely to achieve these goals.

A lack of conceptual clarity about what constitutes an improved care transition makes interstudy comparisons challenging. Notably, close to half of all included studies (*n* = 10) were retrospective in nature and may be prone to confounding by indication bias. Half of the included studies (*n* = 14) focused *primarily* on reducing potentially avoidable transitions, rather than improving them. Researchers have not examined resident- or caregiver-reported outcomes, few have included healthcare professional perspectives, and none have examined transitions from assisted living environments. Last, no existing interventions designed to improve RACF to ED transitions focus on issues such as addressing atypical presentations of older adults during serious changes in health condition (Armstrong, 2015; Chandra et al., 2015) or ageism that can lead to rationing or delayed care for older adults (Adamczyk et al., 2018).

Given the noted methodological and conceptual gaps in the literature, it is challenging to recommend an intervention that can improve RACF to ED interventions. Although standardized documentation forms are frequently used, there is no consensus on critical information to include in these forms, and no authors have evaluated if these interventions improve the quality or accuracy of documentation content. Inconsistent or insufficient documentation practices occur (Tate et al., 2023) and have been linked to adverse outcomes, underdiagnosing, and inappropriate treatments for older adults during transitions in care (Bruce & Suserud, 2005; Morphet et al., 2014). The authors of a recent systematic review (Gettel et al., 2020) also reported a lack of measures to assess critical events during care transitions such as medication errors, duplicative diagnostic testing and procedures, and adverse events. Other literature suggests that documentation interventions should be integrated into electronic recordkeeping and be accompanied with cross-setting education that supports a geriatric focus (McLane et al., 2022; Tate et al., 2023). We recommend that researchers examine the quality and consistency of documentation (vs measuring form uptake and item completion) using more robust, mixed-method approaches. Furthermore, the perspectives of residents and informal caregivers need to be considered to help ensure that essential information (e.g., whether their assistive devices are transported across care settings) is captured (Cummings et al., 2020; McLane et al., 2022).

Geriatric assessments/outreach services and various programs of care were examined in many studies. However, the primary focus of these interventions has been to reduce transitions from RACF to the ED. Results from these studies were mixed and focused on distal outcomes (outcomes that occur late in the transition process or well after the transition has been completed) such as hospital admissions, length of stay, and readmissions. Interdisciplinary approaches are emerging as promising to help reduce unwarranted RACF to ED transitions (Grant et al., 2020). Intentionally adapting these approaches for improving transitions while using established implementation science and feasibility study methods is warranted (Bowen et al., 2009; Damschroder et al., 2009; Waltz et al., 2019). Last, tele- and digital-health services show promise and have increased in uptake during the COVID-19 pandemic. Guidelines for improving digital or telehealth services for people experiencing mental health challenges (Graham et al., 2020) align with the contextual issues (e.g., digital literacy, lack of technical support, and access to technology) identified as important in this review. Implementation strategies such as conducting needs assessments and ensuring supervision and resources pertaining to software training and integration as outlined in Graham et al.’s review (Grant et al., 2020) could be adopted to improve the implementation of tele- and digital-health services.

Current approaches to intervention development do not reflect the advances made in implementation science—as espoused in the Care Transitions Framework (Dy et al., 2015), the Consolidated Framework for Implementation Research, and the Expert Recommendations for Implementation Change tool— to guide planning and implementation of interventions (Damschroder et al., 2022; Waltz et al., 2019). Collectively these frameworks elucidate key strategies for contextualizing and implementing innovations in complex adaptive systems like an RACF or ED. Their application is one means to ensure that intervention implementation reflects the intended approach and is not impeded by factors such as competing provider priorities, insufficient human resources and/or training, and inadequate attention paid to important change management principles (e.g., appropriate application of plan-do-study-act cycles as reported by the Institute for Healthcare Improvement, 2023). These principles are requisite to build skill sets and expertise among stakeholders and thus promote innovation, sustainability, and eventual scale (Damschroder et al., 2022; Waltz et al., 2019). They have been used to successfully implement geriatric screening interventions in EDs and to promote age-friendly care in healthcare in multiple regions (Damschroder et al., 2022; Dolansky et al., 2021). Our findings, derived from identified factors in included studies, support that we may improve intervention development through ensuring that (a) designated leaders are available to support and guide care practice changes, (b) existing policies do not impinge on stakeholders’ ability to enact intervention plans, and (c) proactive discussions and subsequent action takes place to help ensure that planned care changes align with existing care contexts (e.g., by recognizing the limited time that providers have to make decisions in emergency situations and by providing timely access to experts for education and advice). Given the complexity of care transitions (Aase & Waring, 2020) and the need to measure intervention effectiveness more robustly, we recommend that future research should employ mixed-method strategies to more aptly assess implementation and intervention effectiveness. This two-pronged evaluation approach is paramount to differentiate ineffective interventions from potentially promising practices with substandard implementation practices.

Furthermore, researchers are not exploring how care delivery *during* the transition can be improved by interventions (and instead evaluate distal outcomes), and no study employed any frameworks related to transition care (Aase & Waring, 2020) and/or domains of quality to evaluate care, such as Institute of Medicine’s Quality Domains (Agency for Healthcare Research and Quality, 2002; Institute of Medicine (U.S.) Committee on Quality of Health Care in America, 2001). Employing these types of frameworks is needed for developing and applying measures to evaluate the effectiveness of interventions to improve transitions from RACF to the ED across various domains of quality (e.g., safety, person-centeredness, equity; Tate et al., 2022).

### Limitations and Strengths

Our findings may be influenced by publication bias. Only articles published in English were included. As strengths, the review was guided by a theoretical and methodological framework and was conducted in collaboration with health-system decision makers from multiple jurisdictions. Our search strategy was developed in consultation with an academic librarian and we searched five comprehensive databases.

## Conclusions and Implications

This manuscript identifies conceptual, methodical, and measurement shortcomings in the literature seeking to improve RACF to ED transitions, and provides insight into strategies needed to advance this research area. Mixed or nonsignificant results, combined with these research challenges, prevent us from recommending (or discouraging) any intervention to improve RACF to ED transitions. Conceptual clarity and clear, congruent operationalization of an improved transition is needed. We recommend that future research employ established frameworks to clarify the goals for improving transitional care from the viewpoint of various stakeholders, to engage purposefully with stakeholders (e.g., providers, patients) to ensure that proposed innovations are properly contextualized, and to robustly assess innovation success using a range of designs, intervention, and implementation effectiveness metrics.

## Supplementary Material

gnae036_suppl_Supplementary_Materials

## Data Availability

This is a scoping review (no individual level data to report) and therefore pre-registration and data availability requirements are not applicable.
